# Survival case of cardiac strangulation from epicardial pacemaker leads in an adult: A case report

**DOI:** 10.1016/j.radcr.2024.10.019

**Published:** 2024-10-30

**Authors:** Sajjad Haider, Fawaz Mohammed, Ayesha Mahnoor Arab, Sameer Saleem, Jacqueline Dawson Dowe, Akhtar Amin, Aniruddha Singh, Muhammad Shoaib Akbar, Mohammed Kazimuddin, Mohammed Abdul-Waheed

**Affiliations:** a Department of Cardiology, University of Kentucky, Bowling Green, KY 42101, USA; b Department of Medicine, University of Kentucky, Bowling Green, KY 42101, USA; c University of Texas Rio Grande Valley, Edinburg, TX 78539, USA; d Department of Cardiology, Tower Heath, West Reading, PA 19611, USA

**Keywords:** Cardiac strangulation, Epicardial pacemaker lead, Survival case

## Abstract

Cardiac strangulation from leads of an epicardial pacemaker is a rarely reported life-threatening complication. We report the case of a 72-year-old female with history of supraventricular tachycardia status post sino-atrial node ablation with epicardial pacemaker placement who presented to the outpatient setting with shortness of breath. Heart catheterization was performed which showed elevated right and left heart pressures. Compression involving the mid left anterior descending artery and left circumflex artery was noted from an epicardial lead on coronary angiography consistent with cardiac strangulation. The patient was then referred to cardiothoracic surgery for epicardial pacemaker lead removal. The literature has only reported one other survival case of cardiac strangulation in an adult.

## Introduction

Mechanical compression of cardiac muscle can occur when leads of an epicardial pacemaker (EP) wrap around the heart [1]. No set diagnostic and management guidelines exist to recognize cardiac strangulation (CS) in a timely manner often delaying the diagnosis. Case reports are largely limited to the pediatric population [2]. Review of literature has suggested that there is only one other survival case of cardiac strangulation in adults [3]. Herein, we report the only other survival case of CS diagnosed in a seventy-two-year-old female that presented to the outpatient clinic with complaints of shortness of breath.

## Case presentation

**Fig. 1:**
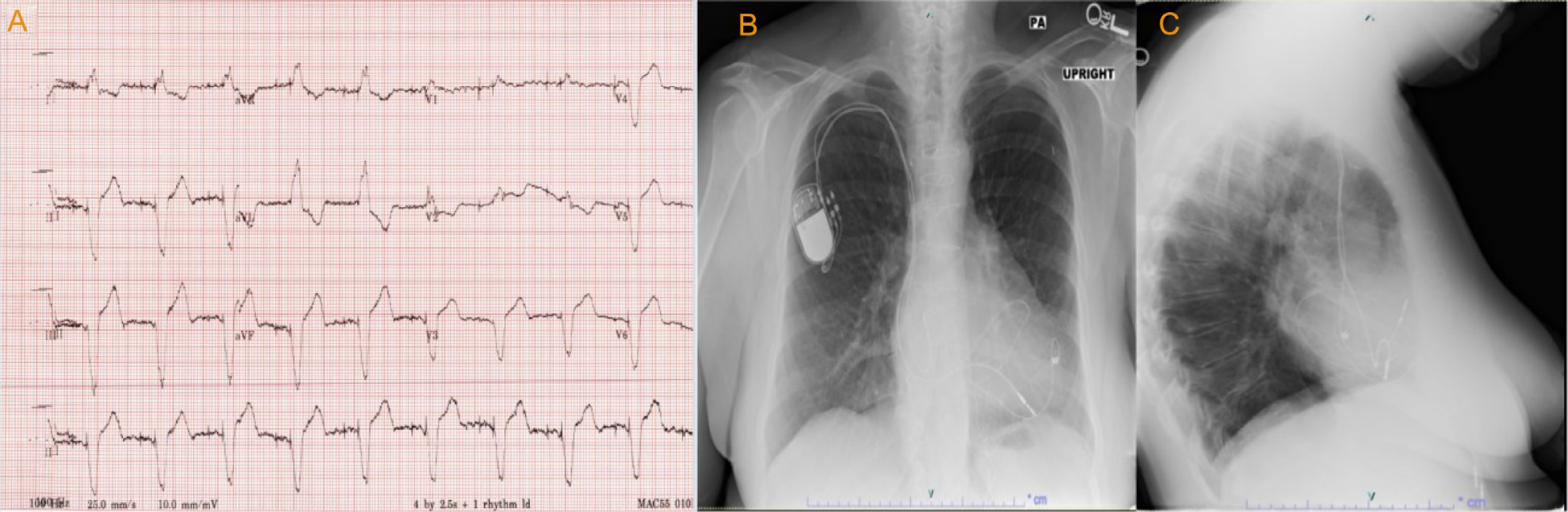
(A) Electrocardiogram demonstrating atrioventricular (AV) paced rhythm, (B) Chest X-ray with posteroanterior and (C) lateral chest views showing pacemaker within right chest with epicardial pacemaker leads with loop around the anterior surface of the heart.

**Fig. 2:**
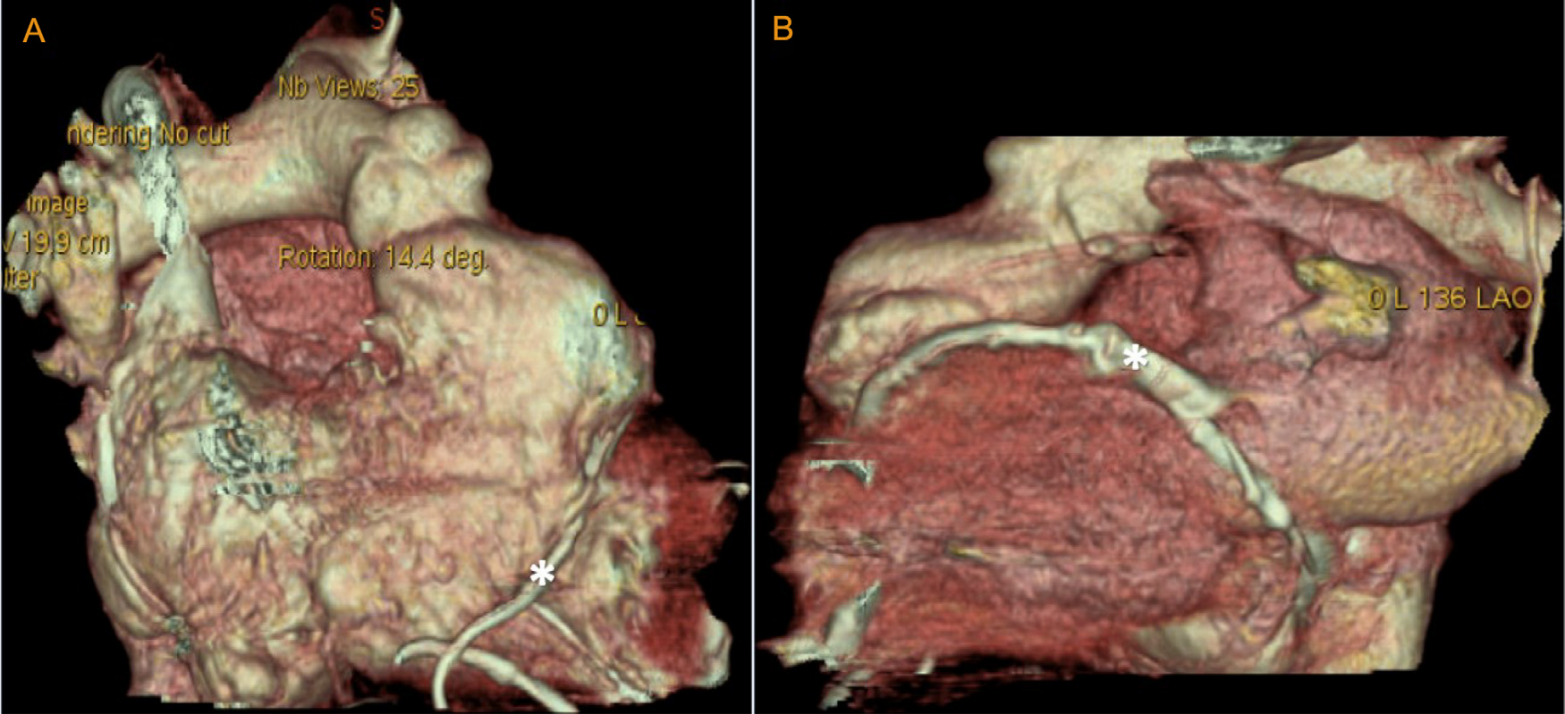
Three-dimensional computed tomography showing (A) epicardial pacemaker lead loop around the (A) anterior and (B) posterior surface of the heart (asterixis).

**Fig. 3:**
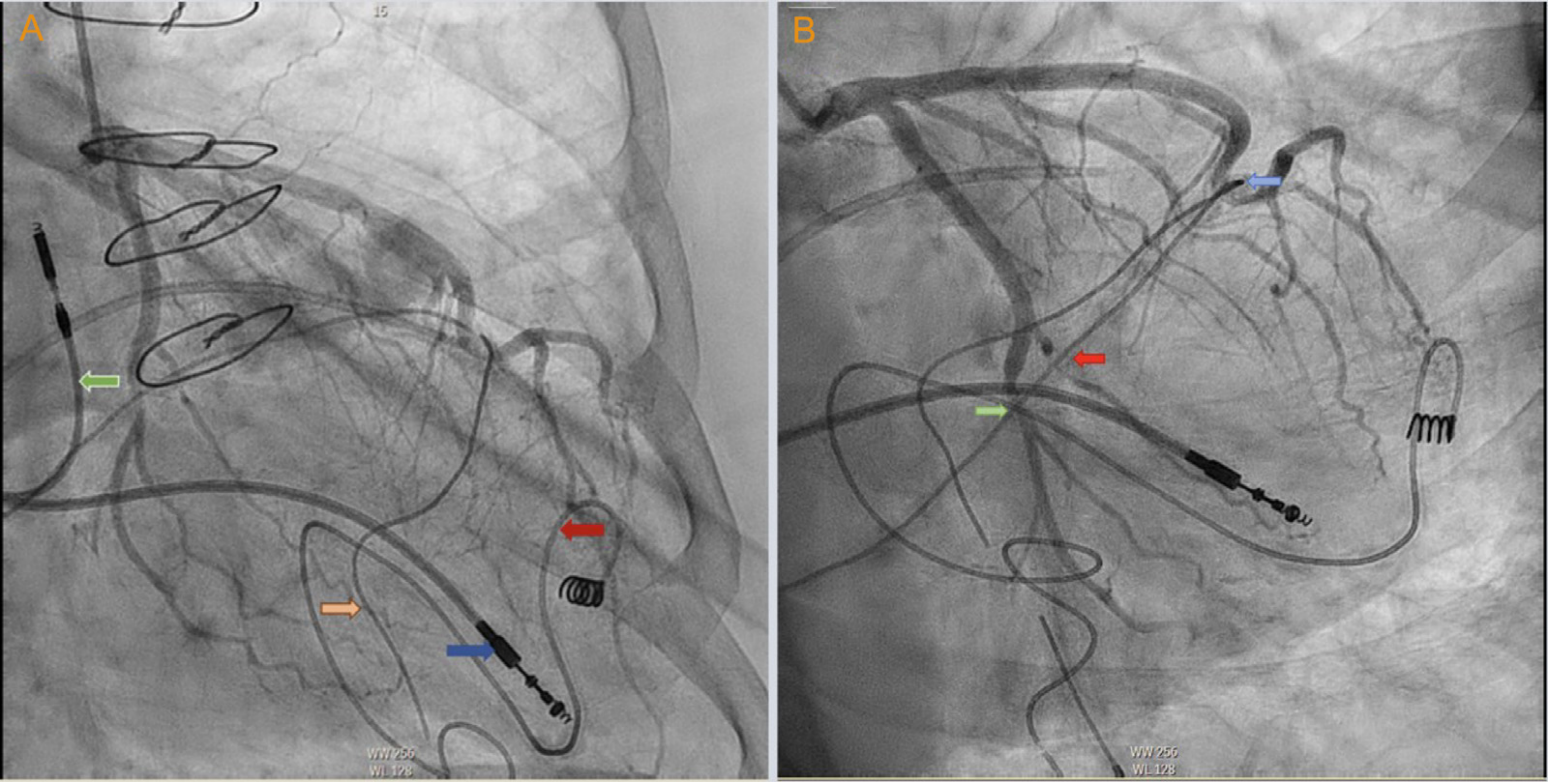
Left heart catherization findings with (A) epicardial atrial lead (blue arrow), epicardial ventricular lead (red arrow), atrial pacemaker lead (green arrow), ventricle pacemaker lead (orange arrow), (B) showing compression of the left anterior descending artery (blue arrow), OM1 (red arrow), left circumflex artery (green arrow) by the epicardial lead.

**Fig. 4:**
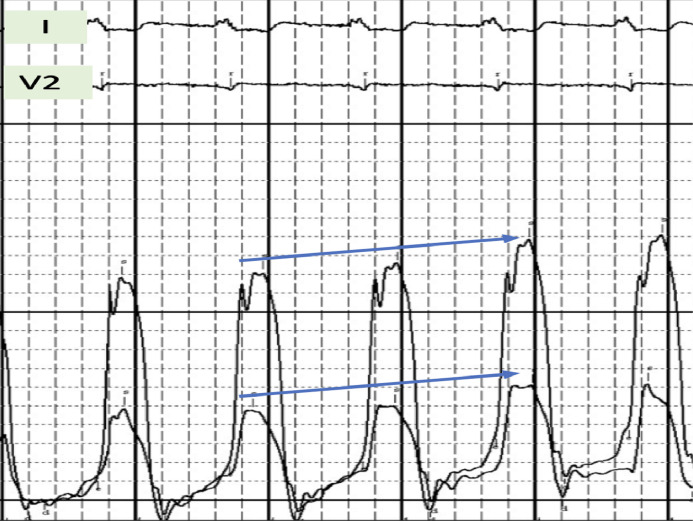
Right heart catherization with simultaneous left and right ventricualr pressure tracing showing concardant pressure changes (arrows); consistent with restrictive cardiomyopathy.

A 72-year-old female presented in the outpatient setting with complaints of shortness of breath that worsened with exertion. She had past medical history of supraventricular tachycardia intervened with sinoatrial node resection followed by EP implantation in the 1980s. She also had past medical history of pericardiectomy followed by dual chamber pacemaker placement for constrictive pericarditis after which the EP generator was extracted. Other medical history was significant for hypertension, hyperlipidemia, restrictive cardiomyopathy, atrial fibrillation and flutter treated with atrioventricular node ablation. She denied symptoms of chest pain, lightheadedness. Physical examination findings were not significant. An electrocardiogram was performed which showed an atrial and ventricle (AV) paced rhythm with a heart rate of 67 beats per minute (Fig. 1A). Previous Chest X-ray showed right ventricular epicardial lead looped around the left ventricle (Fig. 1, Fig. 1). Computed tomography (CT) revealed EP loop encompassing the heart (Figs. 2A and B). With her symptoms of dyspnea, a right and left heart catheterization was performed for further evaluation. Right heart catheterization (RHC) showed right atrial pressure of 10/15 mmHg, mean of 7 mmHg, right ventricular pressure 53/6 mmHg, mean of 14 mmHg, pulmonary artery pressure 53/12 mmHg, mean of 27 mmHg, pulmonary capillary wedge pressure 24/26 mmHg, mean of 27 mmHg, cardiac index 2.74 L/min/m^2^. RHC findings were consistent with restrictive physiology based on simultaneous and right ventricle and left ventricle pressure monitoring (Fig. 3). Coronary angiography revealed compression of the left anterior descending artery, first obtuse marginal branch (OM1) and left circumflex artery by the right ventricular epicardial lead (Figs. 4A and B). The patient was subsequently referred to cardiothoracic surgery for EP lead removal. After extensive discussion with patient regarding the risk associated with surgery for EP lead removal and the presence of restrictive physiology on right heart catheterization; surgery was deferred as the patient wished to be managed with conservatively. Therefore, the patient is being managed with aggressive medical and risk factor modification.

## Discussion

Cardiac strangulation (CS) from leads of an epicardial pacemaker (EP) is a rarely reported devastating complication that was first described in the later 1980s [4]. CS can manifest with an array of symptoms ranging from presyncope, angina, heart failure, myocardial infarction to sudden cardiac death [5]. Asymptomatic cases of CS have also been reported [6]. Symptoms are bought about when epicardial leads mechanically compress structures of the heart impairing cardiac function. Given the ambiguity of symptoms and scarcity of reported cases, no definite algorithmic diagnostic or therapeutic approach has been described making it difficult to diagnose promptly. A total of 3 cases of CS in adults are reported in the literature, with only 1 case that survived [2,3,7]. Our case is the only other case that survived. Kang et al. report a case of CS that was remedied surgically [3]. Surgical removal of EP leads is the only treatment modality for CS to relieve the strangulation with none of the cases reported in the literature managed conservatively. Medical management of CS has not been described. Takeuchi et el. report a study where all but one case of sudden cardiac death was managed surgically with 70% of cases having a favorable outcome [5]. EP leads should be removed promptly when CS is diagnosed. Studies have suggested that lead placement around the anterior pericardial surface or over the main coronary arteries supplying the heart should be avoided to prevent CS [8].

## Conclusion

We report the only other survival case of cardiac strangulation from epicardial leads in an adult. Cardiac strangulation is seen in the pediatric population rarely and is even rarer in the adult population. Surgical intervention with removal of the epicardial leads is the only approach to manage this devastating complication.

## Author contributions

SH, FM, AMA contributed to writing the original draft. SS, JDD, AA, AS, MSA, MK, MAW contributed to reviewing and editing the manuscript for important intellectual content and approval of the final manuscript.

## Patient consent

Written, informed consent for publication of their case was obtained from the patient.
